# Lipid A-Ara4N as an alternate pathway for (colistin) resistance in *Klebsiella pneumonia* isolates in Pakistan

**DOI:** 10.1186/s13104-021-05867-3

**Published:** 2021-12-14

**Authors:** Kiran Iqbal Masood, Seema Umar, Zahra Hasan, Joveria Farooqi, Safina Abdul Razzak, Nazish Jabeen, Jason Rao, Sadia Shakoor, Rumina Hasan

**Affiliations:** 1 Department of Pathology and Laboratory Medicine, Aga Khan University, Karachi, Pakistan; 2 Department of Infection Biology, Faculty Infectious and Tropical Diseases, London School of Hygiene and Tropical Medicine, London, UK; 3 Health Security Partners, Washington, DC 20009 USA

**Keywords:** *Klebsiella pneumonia*, *mcr-1* gene, Antimicrobial drug resistance

## Abstract

**Objectives:**

This study aimed to explore mechanism of colistin resistance amongst *Klebsiella pneumoniae* isolates through plasmid mediated *mcr-1* gene in Pakistan. Carbapenem and Colistin resistant *K. pneumoniae* isolates (n  = 34) stored at − 80 °C as part of the Aga Khan University Clinical Laboratory strain bank were randomly selected and subjected to *mcr-1* gene PCR. To investigate mechanisms of resistance, other than plasmid mediated *mcr-1* gene, whole genome sequencing was performed on 8 clinical isolates, including 6 with colistin resistance (MIC  >  4 μg/ml) and 2 with intermediate resistance to colistin (MIC  >  2 μg/ml).

**Results:**

RT-PCR conducted revealed absence of *mcr-1* gene in all isolates tested. Whole genome sequencing results revealed modifications in Lipid A-Ara4N pathway. Modifications in Lipid A-Ara4N pathway were detected in *ArnA_ DH/FT, UgdH*, *ArnC* and *ArnT* genes. Mutation in *ArnA_ DH/FT* gene were detected in S3, S5, S6 and S7 isolates. *UgdH* gene modifications were found in all isolates except S3, mutations in *ArnC* were present in all except S1, S2 and S8 and ArnT were detected in all except S4 and S7. In the absence of known mutations linked with colistin resistance, lipid pathway modifications may possibly explain the phenotype resistance to colistin, but this needs further exploration.

**Supplementary Information:**

The online version contains supplementary material available at 10.1186/s13104-021-05867-3.

## Introduction

Beta-lactams have always been the backbone of antibiotic regimens targeting infections with Enterobacteriaceae including *K. pneumoniae* [1]. With the increase in resistance to these agents [1, 2] there is interest in using colistin (polymyxin E) for treating such infections. Colistin targets lipopolysaccharides (LPS) and phospholipids present in the outer cell membrane of Gram-negative bacteria, and competitively displaces divalent cations from the phosphate groups of membrane lipids leading to pore formation and disruption of the outer cell membrane, leakage of cytoplasmic contents, and bacterial death.

A worldwide increase in resistance to colistin has been reported globally [3, 4]. Such resistance can either be acquired, or occur as a result of intrinsic factors [5]. Mobile colistin resistance (*mcr*) genes acquired through plasmids were first reported in China in 2015 [6]. The first colistin resistant *E. coli* with *mcr-1* was reported from Pakistan in 2017 [7].

The intrinsic factors contributing to colistin resistance mainly involves the modification of the LPS moiety by the addition of positively charged molecules L-Ara4-N and PEtN [8]. The addition of positively-charged molecules result in the decrease of negative charge in the outer membrane thus reducing its affinity to interact with cationic antibiotics including colistin [9]. Alterations in LPS most commonly occur due to mutations in bacterial genes including mgrB, phoP/phoQ, pmrA, pmrB, pmrC, and crrABC [10–12].Increasing colistin resistance amongst *K. pneumonia*e emphasizes the need to understand mechanisms contributing to such resistance. This study is therefore aimed to explore mechanism of colistin resistance amongst *K. pneumoniae* isolates from Pakistan, and to study the role of plasmid-mediated (*mcr-1* gene) and chromosomal factors contributing to such resistance.

## Main text

### Methods

#### Study subjects

The clinical isolates (n  = 34) were randomly selected from the Aga Khan University Clinical Laboratory strain bank stored at − 80 °C and used anonymously. The source of the selected strains included sputum (n:1), tracheal aspirates (n:9), blood (n:9), urine (n:8), tissue (n:4), pus (n:2) and cerebrospinal fluid (n:1). All the study isolates were pure and were not passaged for more than 3 times.

#### Identification of bacterial isolates and susceptibility testing

The isolates were identified by conventional biochemical methods followed by API-E^®^ (BioMérieux, France). Susceptibilities performed by disc diffusion (Kirby-Bauer) method on Mueller Hinton agar [13] and VITEK2^®^ system (BioMérieux, France) showed resistance against beta-lactams and carbapenems (imipenem/meropenem).

Colistin susceptibility was performed using broth micro-dilution according to Clinical and Laboratory Standards Institute (CLSI) guidelines, M07-A10, 2019 [14]. Minimum Inhibitory Concentrations (MIC) were performed in 96-well polystyrene microtitre plates using Cation-Adjusted Mueller Hinton Broth and colistin sulfate powders (Sigma-Aldrich, Inc.) Colistin concentration of 0.03–16.0 µg/ml was used [15]. ATCC *E. coli* 25922, ATCC *P. aeruginosa* 27853 and NCTC *E. coli* 13486 were used as controls. Results were read and interpreted after 16–20 h at 35 °C using colistin cut-offs (≤ 2 µg/ml  =  intermediate;  ≥ 4 µg/ml  =  resistant) for Enterobacteriales [14].

#### DNA extraction

The selected isolates (n  = 34) were inoculated into Brain heart infusion for 24 h. 1 ml of the broth was used for DNA extraction as per QIA^®^amp DNA extraction kit (QIAGEN, USA) manufacturer’s protocol. The quality of the extracted DNA was examined using Qubit v.2.0 fluorometer (Life Technologies, USA). The extracted DNA was stored at − 80 °C till further processing.

#### *mcr-1* gene real-time PCR

Real‐time PCR was performed on the extracted DNA in duplicate. 20‐μl reactions were set up containing Platinum^®^ SYBR^®^ Green qPCR Supermix‐UDG (Invitrogen), 150 nm forward and reverse primers and 2 μl of DNA on a BioRad CFX 7500 thermal cycler. Sequence specific primers were used for *mcr-1* [16] gene and the house keeping gene *rho*, [17]. Primer sequences are provided in Additional file 1: Table S1. NCTC 13846 *E. coli* was used as positive control.

## Whole genome sequencing

Eight *K. pneumoniae* (CRKP1-CRKP6) strains were selected for whole genome sequencing (WGS) with six resistant and two intermediate to colistin. We had clinical data on only 4 isolates: one colistin intermediate isolate failed microbiological clearance for 3 years despite treatment, while three colistin resistant isolates achieved microbiological clearance between 7 and 11 days.

DNA was shipped to Eurofins scientific SE, Luxembourg for sequencing. Sequencing was carried out on the Illumina NovaSeq 6000 platform using 2 × 150 Sequence mode. WGS was performed with the genome coverage of 99%. Genetic analysis of reads was done using the KmerGeni tool which generated an approximate of 5.1 Mbp contigs. The de novo assemblies were evaluated by QUAST and Icarus tools. The evaluation was based on the alignment of the de novo assemblies on the reference genome (HS11286). The contigs were annotated with RAST to look for resistance genes.

## Data submission

The raw sequences generated after sequencing were submitted to NCBI-SRA website under the accession numbers SAMN16684225–SAMN16684230. This Whole Genome Shotgun project genome assemblies were deposited at DDBJ/ENA/GenBank under the accession JAEMV(N/O/P/Q/R/S)000000000. The version described is JAEMVX010000000. The complete data details are available in the BioProject PRJNA674952.

## Data analysis

The raw Illumina reads were passed through quality check using FASTQC and then assembled with SPAdes-3.13.0 software using spade and plasmid spade scripts to generate raw chromosomal and plasmid contigs. The generated assembly quality assessment was conducted using QUAST (http://bioinf.spbau.ru/quast). Continuous chromosome sequence was generated by overlapping raw contigs assembly against the reference genome by abacas.1.3.1 perl script that closed gaps on shotgun assembled contigs against the reference genome based on alignment between assembly and reference to identify syntenies of contigs with the reference.

Multi-Locus Sequence Typing (MLST) was determined using *K. pneumoniae* Sequence Typing web-based tool (PasteurMLST) (https://bigsdb.pasteur.fr/). The MLST was performed using the seven housekeeping genes (gapA, infB, mdh, pgi, phoE, rpoB and tonB) according to the protocol described by Diancourt et al. [18]. RAST server (https://rast.nmpdr.org/) and Center for Genomic Epidemiology server (www.cbs.dtu.dk/services) were used for Chromosomal and Plasmid sequence annotations and downstream analysis.

## Phylogenetic analysis

The phylogenetic inference was done by aligning the eight isolates genome with the reference using MegaX software (https://www.megasoftware.net/). The aligned sequences were then converted into a Phylodendogram using the software package Clonal Frame version 1.1. The dendogram was then estimated under the maximum likelihood (ML) principle in PhyloXML (http://www.phyloxml.org/). The tree was colored and edited using FigTree (http://tree.bio.ed.ac.uk/software/figtree/). The tree was further magnified to show closely related genome using SNP (single nucleotide polymorphism) cluster generated by Pathogen detection Browser (https://www.ncbi.nlm.nih.gov/pathogens/isolates/).

### Results

#### *mcr-1* gene PCR

A total of 34 colistin resistant *K. pneumoniae* isolates were included. These strains were investigated for the presence of *mcr-1* gene. However, PCR conducted revealed absence of *mcr-1* gene in all isolates tested.

#### Alternate mechanisms involved in colistin resistance

WGS was performed to further understand the mechanism of colistin resistance. Mutations in genes involved in lipid-A and Ara-4 N pathways were revealed through a variant analysis of non-synonymous single nucleotide variants (ns-SNVs Table 1).

**Table 1:** Lipid A pathways related variants identified in colistin-resistant *Klebsiella pneumoniae* isolates

Isolates	MIC	GenBank accession	Lipid A modification		Lipid A-Ara4N pathway (polymyxin resistance)			
			PagP	PhoR	*ArnA_ DH/FT*	*UgdH*	*ArnC*	*ArnT*
S1	0.25	JAEMVQ000000000.1	I189F	A424_V425insSerAla	–	N354D	–	A55G
				L65F				
S2	0.5	JAEMVP000000000.1	I189F	A424_V425insSerAla	–	V17I	S10fs	A55G
S3	4	JAEMVA000000000	I189F	L65F	T185A	–	–	A55G
S4	8	JAEMVS000000000.1	I189F	L65F	–	N354D	S10fs	–
S5	4	JAEMVR000000000.1	I189F	A424_V425insSerAla	T185A	N354D	S10fs	A55G
				L65F	S18A		S10fs	
S6	16	JAEMVO000000000.1	I189F	A424_V425insSerAla	T185A	N354D	S10fs	A55G
				L65F	S18A			
S7	16	JAEMVN000000000.1	I189F	A424_V425insSerAla	L260I	A376V	S10fs	–
				M45I	D205N	N354D		
S8	≥ 16	JAEMVB000000000	I189F	L65F	–	V17I	–	A55G

*MIC* minimal inhibitory concentrations. The table represents various mutations (amino acid) found upon whole genome sequencing analysis of eight *Klebsiella pneumonia* strains isolated from clinical samples

The mutations detected in PagP gene (I189F) were present in all isolates. In Pho R gene, mutations identified included: A424_V425insSerAla present in all isolates except S3, S4 and S8; L65F present in all isolates except S2 and S7. Additionally, M45I mutation was also identified in isolate S7.

Modifications in Lipid A-Ara4N pathway were detected in *ArnA_ DH/FT, UgdH, ArnC* and *ArnT* genes. *ArnA_ DH/FT* included mutations T185A, S18A, L260I and D205N. Mutations detected in *UgdH* were N354D, V17I, N354D. Mutation detected in *ArnC* was S10fs and in *ArnT* was A55G.

Mutations in *ArnA_ DH/FT* gene were detected in S3, S5, S6 and S7 isolates. *UgdH* gene modification was found in all isolates except S3, mutations in ArnC were present in all except S1, S2 and S8 and ArnT were detected in all except S4 and S7.

#### Multi locus sequence typing of *K. pneumoniae*

MLST revealed that the isolates belonged to ST37 (n=1), ST147 (n=3), ST14 (n=1), ST 11 (n=1), ST39 (n=1) and ST 17 (n=1) sequence types (Table 2).

**Table 2:** The general genetic characteristics of the *Klebsiella pneumoniae* strains

Samples	No. of contigs	Total length of assembly	N50	GC content (%)	Multi-locus sequence typing (MLST)	Plasmids names	Percent identity against plasmid
S1	99	5,817,465	1,00,468	56.63	14	ColKP3, IncFIB(K), IncFIB(Mar), IncFII, IncHI1B, IncR	100, 100, 99, 100, 100, 100
S2	53	5,166,762	8,02,570	57.35	11	IncA/C2, IncFIB(pQil), IncFII(K)	100, 100, 100
S3	87	5,709,294	1,85,631	56.96	147	Col440I, ColRNAI, IncFIB(pQil), IncFII(K), IncL/M(pOXA-48), IncR, IncX4	96, 96, 96, 97, 100, 100, 100
S4	88	5,699,223	1,67,147	56.72	37	ColKP3, FIA(pBK30683), IncFII(K), IncHI1B	100, 97, 95, 99
S5	254	5,993,514	1,00,468	56.63	147	Col440I, ColKP3,ColRNAI, IncFIB(pKPHS1, IncFIB(pQil), IncFII(K), IncL/M(pOXA-48), IncR, IncX4	96, 100, 96, 98, 100, 97, 100, 100, 100
S6	72	5,758,675	2,98,382	56.99	147	ColRNAI, IncFIB(pQil), IncFII(K), IncL/M(pOXA-48), IncR	96,100, 97, 100, 100
S7	97	5,408,166	2,59,061	57.33	39	Col440I, IncFIB(pQil), IncFII(K)	100, 100, 97
S8	122	5,742,261	2,63,702	56.95	17	IncFIB(K), IncFIB(pKPHS1), IncFIB(pQil), IncFII(K), IncX3	99, 98, 100, 97, 100

*No. of contigs* number of contiguous data; *N50* sequence length at 50%, *GC content* glycine cystine content; *MLST* multi-locus sequence typing. The most common MLST sequence strain isolated was ST147 (n  = 3)

#### Phylogenetic analysis

**Fig. 1:**
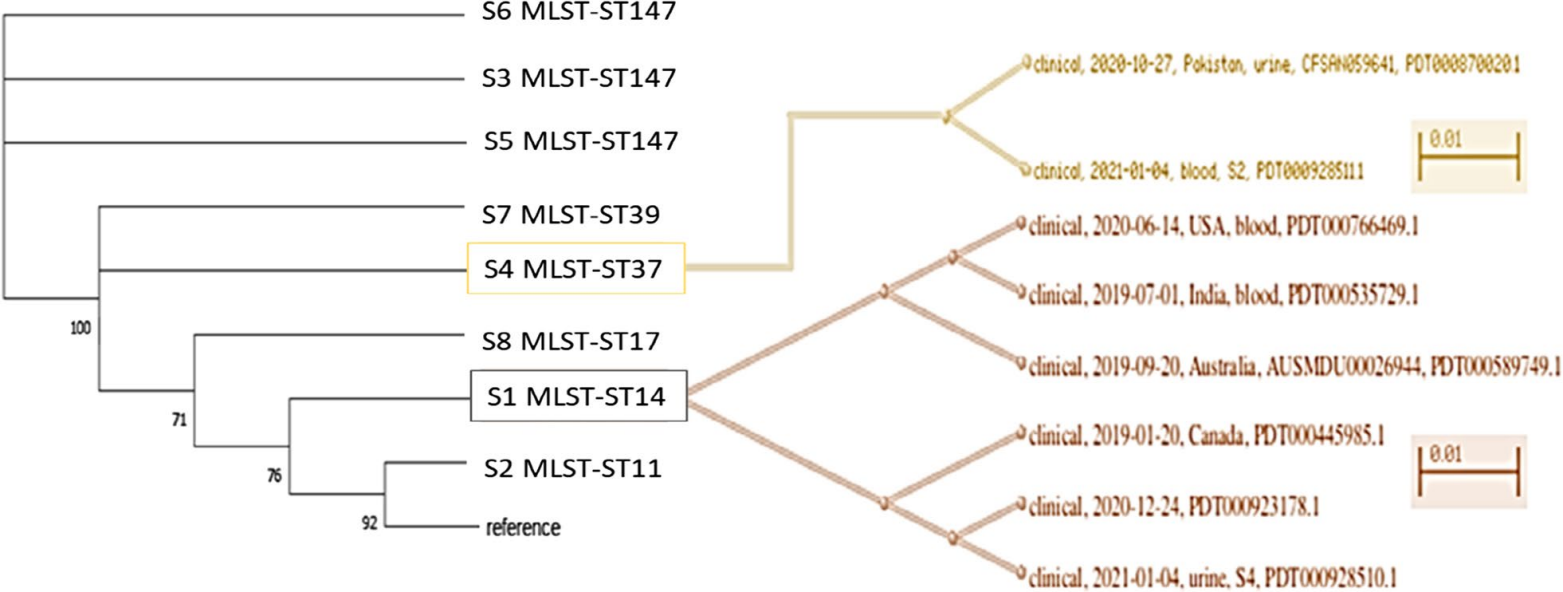
Dendrogram colistin-resistant—*Klebsiella pneumoniae* isolates, along with magnified sub-clades for S1 and S4 based on SNP cluster was plotted using Pathogen Detection Browser

The dendrogram was plotted (n = 41,172 as per 6th March 2021) using NCBI Pathogen Detection database (Fig. 1). S3, S5 and S6 (ST147) did not cluster with the reference strain, however, S4 (ST37) was the closest, followed by S2 (ST11) and then S1 (ST14) and S8 (ST17).

In addition to the above, clustering with other sequences deposited in the database was also observed. S4 clustered with clinical strain CFSAN059641 isolated in 2020 in Pakistan and also with the other clinical strains deposited (PDT0009285111). S1 clustered with clinical strains isolated in USA (2020), and also with others from Australia, Canada and India (2019).

### Discussion

Although novel treatment approaches are being explored [20–23], increasing prevalence of colistin resistant *K.* *pneumoniae*, continue to pose a serious global threat.

In Pakistan *mcr-1* gene has been detected in 23.3% *E. coli* (n  = 120) and 40% of *K. pneumoniae* (n  = 60) [24]. Our study however revealed absence of *mcr-1* gene in all isolates tested. These findings are consistent with earlier studies also reporting absence of *mcr-1* gene in colistin resistant clinical study isolates [25] and suggest presence of factors other than *mcr-1* gene contributing to colistin resistance in these isolates.

Alternative mechanisms suggested for colistin resistance include: decreased drug permeability by porin loss, resistance to antibiotic penetration through bio-film formation [26] and mutations leading to alteration in antibiotic binding sites and efflux pump [27]. In our isolates WGS showed the modifications in PagP and PhoR genes of lipid A pathway. Mutations in PagP has been shown to be associated with colistin resistance [28, 29]. Also, mutations in the phoB-phoR operon have shown to contribute to the anti-microbial resistance by downregulation of PhoE [30]. Mutations found in genes of Lipid A-Ara4N pathway including *Arn_DH/F*, *ArnC*, *ArnT* have been reported in *K. pneumonia* [28]. We additionally detected mutations in *UgdH* gene of Lipid A-Ara4N pathway which has been shown to be implicated in colistin resistance in *E. coli* [31]. Gram negative bacteria develop resistance against cationic antimicrobial peptides by masking negative charges of the lipid A phosphate substituent through the addition of L-Ara4N positively charged-moieties. Briefly, L-Ara4N is transferred to lipid A by a lipid carrier in a reaction catalysed by ArnT. The synthesis of lipid carrier linked to L-Ara4N is catalyzed by UDP-Glc 6-dehydrogenase (Ugd), ArnA, ArnB, ArnC, and ArnD [32]. This pathway is well-explained in studies in *E. coli* and *S. enterica *sv.* typhimurium* [33]. It is important to note that the mutations found in Lipid A and L-Ara4N pathway were present in all isolates comprising of 6 colistin resistant and 2 colistin intermediate strains. One of our colistin intermediate isolate with mutations in both Lipid A and A-Ara4N pathway, failed to achieve microbiological clearance over 3 years, despite treatment. There is inherent variability in testing methods for colistin susceptibility, hence the susceptible category has been removed by CLSI [14]. Colistin monotherapy is also discouraged to avoid excessive reliance on even the currently recommended broth dilution susceptibility testing method. Hence, learning more about molecular mechanisms of colistin resistance might be helpful for taking clinical decisions in future.

The MLST done showed that eight *K. pneumoniae* isolates belonged to 6 different sequence types (ST11, ST14, ST17, ST37, ST39 and ST147) hence pointing towards the presence of considerable genetic diversity among them. Previous studies have shown that ST11 and ST14 have been detected amongst clinical isolates from Pakistan [25]. Globally, ST11 has been reported in Korea [34], Tunisia [35], and Egypt and linked with the presence of CTX-M gene [3]. ST 14 has been shown to be circulating in Dubai [36], India [37], Pakistan [25] and described to carry NDM-1 and CTX-M [38]. ST 17 are also reported to be mostly extended spectrum beta lactamases (ESBL) carrying clones [39]. ST 147 detected has been shown globally to be linked with resistance [15, 40, 41]. ST37 has been reported with carbapenem resistance and ST39 [42] with ESBLs.

This study highlights the significant challenges posed by multi-drug resistant *K. pneumoniae* strains to global health and emphasizes the need to identify factors contributing to resistance towards their better treatment and control.

## Limitations

Although the study highlights mechanism of colistin resistance alternate to *mcr-1* gene in clinical isolates in Pakistan, it can be further strengthened by increasing sample size and by performing functional studies to validate the role of mutations found in Lipid A and L-Ara4N pathway in contributing colistin resistance.

## Supplementary Information

**Additional file 1: Table S1.** Primers and probe sequences to target the plasmid-mediated colistin resistance (*mcr-1*) and housekeeping gene *rho*. **Figure S1.** Amplification of* mcr-1* gene in a positive control NCTC 13846 *E. coli* strain. DNA was extracted from the pure culture of NCTC 13846 *E. coli* strain using DNA extraction kit (Qiagen) as per manufacturers instruction. Extracted DNA was subjected to PCR using sequence specific primers of *mcr-1* gene. The figure shows the amplification of *mcr-1* gene in the positive control used in the experiment. PC denotes positive control and NTC denotes non-template control.

## Data Availability

The datasets used and/or analyzed during the current study are available from the corresponding author on reasonable request.

## Abbreviations

DNA: Deoxyribonucleic acid
ESBL: Extended spectrum beta lactamases
LPS: Lipopolysaccharide
MDR: Multi drug resistant
*mcr*1: Mobile colistin resistance 1
MLST: Multi-locus sequence typing
NCTC: National collection of type culture collection strain
WGS: Whole genome sequencing
