# Supplementary material for: Lipid A-Ara4N as an alternate pathway for (colistin) resistance in Klebsiella pneumonia isolates in Pakistan
Source: BMC Res Notes. 2021 Dec 14;14:449. doi: 10.1186/s13104-021-05867-3 (PMC8670247; doi:10.1186/s13104-021-05867-3)
Supplement: Supplementary file 1 — Additional file 1: Table S1. Primers and probe sequences to target the plasmid-mediated colistin resistance (mcr-1) and housekeeping gene rho. Figure S1. Amplification of mcr-1 gene in a positive control NCTC 13846 E. coli strain. DNA was extracted from the pure culture of NCTC 13846 E. coli strain using DNA extraction kit (Qiagen) as per manufacturers instruction. Extracted DNA was subjected to PCR using sequence specific primers of mcr-1 gene. The figure shows the amplification of mcr-1 gene in the positive control used in the experiment. PC denotes positive control and NTC denotes non-template control. [file 13104_2021_5867_MOESM1_ESM.docx]

**Lipid A-Ara4N as an alternate pathway for (colistin) resistance in Klebsiella pneumonia isolates in Pakistan**

Table 1: Primers and probe sequences to target the plasmid-mediated colistin resistance (*mcr-1*) and housekeeping gene *rho*

| ***mcr-1* PRIMER/PROBE** | Sequence |
| --- | --- |
| PE_F1 | GCAGCATACTTCTGTGTGGTAC |
| PE_R1 | ACAAAGCCGAGATTGTCCGCG |
| PE_PROBE 1 | FAM - GACCGCGACCGCCATCTTACC-TAMRA |
| PE_F2 | GGGTGTGCTACCAAGTTTGCTT |
| PE_R3 | TATGCACGCGAAAGAACTGGC |
| PE_PROBE | FAM - GCGCTGATTTTACTGCCTGTGGTG-TAMRA |
| **Housekeeping gene rho** |  |
| Forward | AACTACGACAAGCCGGAAAA |
| Reverse | ACCGTTACCACGCTCCATAC |

**S1: Amplification of *mcr-1* gene in a positive control NCTC 13846 *E. coli* strain.**

**
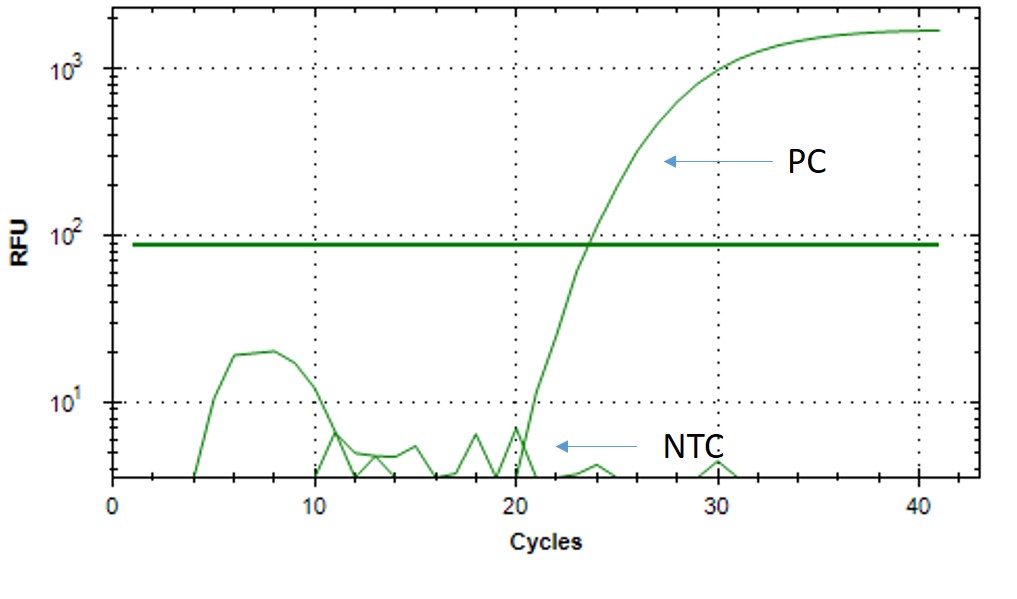
**

**Fig S1. Amplification of *mcr-1* gene in a positive control NCTC 13846 *E. coli* strain**. DNA was extracted from the pure culture of NCTC 13846 *E. coli* strain using DNA extraction kit (Qiagen) as per manufacturers instruction. Extracted DNA was subjected to PCR using sequence specific primers of *mcr-1* gene. The figure shows the amplification of *mcr-1* gene in the positive control used in the experiment. PC denotes positive control and NTC denotes non-template control.
